# Comparison of Capsular Closure and Capsulectomy in Primary Total Hip Arthroplasty Using the Direct Anterior Approach

**DOI:** 10.1055/s-0046-1818602

**Published:** 2026-04-22

**Authors:** Bruno Dutra Roos, Milton Valdomiro Roos, Ezequiel Moreno Ungaretti Lima, Mauricio Domingos Betto, Juan Victor Guzman Gonzales, Henrique Emanuelli Della Méa

**Affiliations:** 1 Hip Surgery Service, Hospital Ortopédico de Passo Fundo, Passo Fundo, RS, Brazil

**Keywords:** arthroplasty, replacement, hip, comparative study, joint capsule, prospective studies, treatment outcome, artroplastia de quadril, capsula articular, estudo comparativo, estudos prospectivos, resultados do tratamento

## Abstract

**Objective:**

To compare the clinical, radiographic, and complication outcomes of patients undergoing capsular repair or resection during total hip arthroplasty (THA) using the anterior approach.

**Methods:**

The present prospective cohort study included 232 patients submitted to THA at a tertiary hospital, who were divided according to exposure. One group of subjects underwent THA with capsular preservation and closure, and the other underwent THA with capsular resection (capsulectomy). Clinical assessment included the Harris Hip Score (HHS), evaluation of hip range of motion, and identification of symptoms indicative of iliopsoas tendinitis. Additionally, a radiological assessment determined the presence of heterotopic ossification, and we evaluated any postoperative complications.

**Results:**

There was no statistically significant difference in HHS and hip range of motion. No patient from the capsular closure group presented complications. In the capsulectomy group, 7 (5.20%) subjects presented with heterotopic ossification, including 1 case classified as Brooker grade 3, besides 3 (2.25%) cases of iliopsoas tendinitis and 1 (0.75%) case of prosthesis dislocation.

**Conclusion:**

Capsular closure in THA using the direct anterior approach resulted in a lower incidence of iliopsoas tendinitis, heterotopic ossification, and complications, such as prosthesis dislocation, compared with the capsulectomy group.

## Resumo

**Objetivo:**

Comparar os resultados clínicos, radiográficos e complicações de pacientes submetidos à cirurgia de reparo ou ressecção capsular em artroplastia total de quadril (ATQ) pela via anterior.

**Métodos:**

Estudo de coorte prospectivo com uma amostra de 232 pacientes submetidos à cirurgia de ATQ em um hospital terciário. Os pacientes foram divididos, conforme a exposição, em um grupo submetido à ATQ com preservação e fechamento capsular e outro grupo submetido à ATQ com ressecção capsular (capsulectomia). Clinicamente, os pacientes foram avaliados de acordo com o Harris Hip Score (HHS), arco de movimento do quadril e presença de sintomas de tendinite do iliopsoas. Radiograficamente avaliou-se a presença de ossificação heterotópica. Avaliou-se também a ocorrência de complicações pós-operatórias.

**Resultados:**

Não se observou diferença estatisticamente significativa em relação ao escore clínico do HHS e arco de movimento do quadril. No grupo fechamento capsular não foram observadas complicações. No grupo capsulectomia, observou-se 7 casos (5,20%) de ossificação heterotópica, com um caso classificado como grau 3 de Brooker, além de encontramos 3 (2,25%) casos de tendinite do iliopsoas e 1 (0,75%) caso de luxação de prótese.

**Conclusão:**

O fechamento capsular na ATQ por via anterior direta demonstrou menor incidência de tendinite do iliopsoas, ossificação heterotópica e complicações como luxação de prótese, em comparação ao grupo capsulectomia.

## Introduction

Total hip arthroplasty (THA) is a widely used surgical procedure that improves the quality of life in patients with degenerative diseases of the hip joint. This joint consists of the congruence between the femoral head and the acetabulum, stabilized by structures such as the articular capsule, round ligament, acetabular labrum, and adjacent musculature. The articular capsule contains proprioceptive and nociceptive receptors. It plays a key role in joint stability, acting as a protective mechanical barrier against impacts and excessive loads. 1 2 In recent years, the direct anterior approach (DAA) has gained popularity due to its benefits compared with other surgical techniques, including less tissue damage, faster recovery, and better aesthetic results. However, its adoption requires proper patient selection and a learning curve for the surgeon. 3 4 5

Some authors have recommended anterior capsule repair during THA using DAA based on anatomical and biomechanical studies that demonstrate the significance of the capsule in joint stability. Partial resections may increase the risk of instability, as evidenced in cadaveric models. 6 7

Therefore, the present study aimed to analyze and compare the clinical outcomes, hip range of motion, iliopsoas tendinitis symptoms, radiographic results, and complications in patients undergoing capsular repair and resection in THA using DAA.

## Methods

The present prospective cohort study initially selected 332 adult patients of both sexes from a tertiary hospital. The inclusion criterion was having undergone THA using DAA performed by a single surgeon experienced in this technique, who was a member of the research team, between October 1, 2021, and June 1, 2024.

Exclusion criteria included patients who underwent THA for acetabular and proximal femur fractures, revision THAs, had a current infection, needed bone reconstructions, had a follow-up duration of less than 1 year, or did not consent to participate in the research.

Based on the established criteria, 232 patients satisfied the eligibility requirements for the study. Eligible study participants were divided into two groups. Group 1 underwent primary THA using DAA with capsular preservation and closure, while group 2 underwent primary THA using DAA with joint capsule resection (capsulectomy).

The clinical evaluation of the participants was performed using the Harris Hip Score (HHS) pre- and postoperatively at 3 months, 1 year, and annually thereafter. Preoperatively and at the last postoperative visit, the operated hip flexion and external rotation were measured using a goniometer. The objective was to assess whether there was a difference in hip flexion and external rotation between the groups. At the last postoperative evaluation, patients were also evaluated for persistent groin pain associated with physical examination revealing pain on resisted hip flexion, to determine the presence of symptoms suggestive of iliopsoas tendinitis. In the presence of these symptoms, a magnetic resonance imaging (MRI) was requested for diagnostic confirmation.

At the final follow-up visit, radiographic evaluation was performed to assess the presence of heterotopic ossification according to the Brooker et al. ^8^ classification. In addition, complications requiring hospital readmission or not were assessed.

All patients individually signed the Informed Consent Form. Based on Resolution 466/2012 of the Brazilian National Health Council, this study complied with the ethical principles of beneficence, non-maleficence, justice, and autonomy. The Ethics and Research Committee of the CEP/CONEP system approved this study under number CAAE: 75628123.7.0000.5342.

Statistical analyses were performed using the IBM SPSS Statistics for Windows (IBM Corp.) software, version 24.0, with the propensity score matching (PSM) extension. Comparisons of categorical variables used Fisher's exact test or Pearson's Chi-squared test. Owing to violations of normality, as assessed by the Kolmogorov-Smirnov test, comparisons between numerical variables were conducted using the non-parametric Mann-Whitney U and Friedman tests. The minimum significance level was 5%.

### Surgical Technique

The patient is positioned supine on a conventional radiolucent table. The skin incision typically begins approximately 2 cm distal and 2 cm lateral to the anterior superior iliac spine, and it extends distally and laterally toward the lateral border of the patella for a length of 6 to 10 cm. Following skin incision and hemostasis of the subcutaneous tissue, the space between the sartorius muscle, which often has a tendinous appearance, and the tensor fasciae latae (TFL) muscle is identified. A Hohmann retractor with a cobra-shaped head is placed superior to the femoral neck for lateral retraction of the gluteus medius. Next, a Meyerding retractor medially retracts the reflected head of the rectus femoris. The ascending branches of the lateral circumflex femoral artery, located beneath the deep fascial layer of the TFL, are carefully dissected and ligated or coagulated to minimize intraoperative bleeding. A second Hohmann retractor is positioned medially to the femoral neck to retract the TFL. Subsequently, the medial Meyerding retractor is removed, and a third Hohmann retractor is placed on the anterior column of the acetabulum to expose the articular capsule. At this stage, either resection of the anterior articular capsule is performed or an L-shaped capsular flap is created along the medially based intertrochanteric line, preserving the anterior capsule. The procedure then proceeds in a standard fashion with implantation of the acetabular and femoral components. Articular capsular closure, if required, uses continuous 1-0 polyglycolic sutures. 9 10 11 12

## Results

The patients' ages ranged from 18 to 83 years, with a mean age of 61.3 ± 12.9 years. Of the 232 participants, 138 (59.5%) were male, and 94 (40.5%) were female. The majority of patients were diagnosed with coxarthrosis, accounting for 192 (82.8%) cases. Grade-3 osteoarthritis was the most prevalent, observed in 191 (82.3%) patients, followed by grade 2 in 36 (15.5%) patients, and grade 1 in 5 (2.2%) patients ( Table 1 ). The average follow-up time was 19.3 months (ranging from 12 to 34 months). Of the total, 213 (91.8%) arthroplasties were uncemented, 15 (6.4%) were hybrid, and 4 (1.7%) were cemented. Group 1 (capsular closure) consisted of 99 patients (42.7%) with an average follow-up of 14.2 months (12–18 months), and group 2 (capsulectomy) consisted of 133 patients (57.3%) with an average follow-up of 24.4 months (14–34 months).

**Table 1:** Sample characterization per total hip arthroplasty group as median (interquartile range) values and absolute numbers (%)

Variables	Group 1	Group 2	*p* -value
**Age**	62.0 (55.0-70.0)	63.0 (54.3-70.0)	0.804**
**Sex**			
Female	35 (37.2)	59 (62.8)	0.179 ^¥^
Male	64 (46.4)	74 (53.6)	
**Osteoarthritis etiology**			
Coxarthrosis	81 (42.2)	111 (57.8)	0.165 ^ɸ^
ANFH	5 (45.5)	6 (54.5)	
Sequela from DHD	2 (22.2)	7 (77.8)	
Acetabular protrusion	5 (55.6)	4 (44.4)	
Leg-Perthes	0 (0.0)	3 (100.0)	
Sequelae of a fracture	6 (75.0)	2 (25.0)	
**Preoperative osteoarthritis degree**			
1	2 (40.0)	3 (60.0)	0.236 ^ɸ^
2	20 (55.6)	16 (44.4)	
3	77 (40.3)	114 (59.7)	

**Abbreviations:** ANFH, acetabular necrosis of the femoral head; DHD, dysplastic hip disease.

**Notes:** *Chi-squared test; **Mann-Whitney U test; ¥ Fisher's exact test; ɸ Pearson's Chi-squared test. **Group 1:** Primary total hip arthroplasty using the direct anterior approach with capsular preservation and closure. **Group 2** : Primary total hip arthroplasty using the direct anterior approach with resection of the articular capsule (capsulectomy).

In group 1, the mean HHS was 52.5 points (41.5–62.5) preoperatively, increasing to 92.5 (80.7–99.9) at 3 months postoperatively and to 97.3 (94.6– 99.8) at the final follow-up. Preoperatively, the mean hip flexion range of motion was 68.9 degrees (50.0–90.0 degrees), and the mean external rotation was 6.0 degrees (0.0–15.0 degrees). At the last follow-up, the mean hip flexion range of motion was 100.0 degrees (90.0–110.0 degrees), and the mean external rotation was 32.0 degrees (10.0–45.0 degrees). There were no diagnoses of iliopsoas tendinitis, radiographic evidence of heterotopic ossification, or readmissions due to prosthetic instability, infection, or pulmonary embolism.

In group 2, the mean HHS was 52.4 points (41.0– 62.8) before surgery, increasing to 92.4 (77.9–99.9) in 3 months and to 97.0 (94.2–100.0) at the last follow-up. Preoperatively, the mean hip flexion range of motion was 69.7 degrees (50.0– 90.0 degrees), and the mean external rotation was 7.2 degrees (0.0– 20.0 degrees). At the last follow-up, the mean hip flexion range of motion was 100.3 degrees (90.0– 110.0 degrees) and the mean external rotation was 34.0 degrees (5.0–45.0 degrees). Fourteen subjects had postoperative complications, all from the THA group without capsular closure. Therefore, it was not possible to apply analytical statistical tests due to the presence of zero cases in the THA group with capsular closure. Three cases (2.25%) of iliopsoas tendinitis were observed. Radiographically, there were 7 cases (5.20%) of heterotopic ossification (6 Brooker grade 1 and 1 Brooker grade 3). Regarding the need for readmission, 1 case (0.75%) of non-recurrent prosthetic dislocation, 1 case of pulmonary embolism (0.75%), and 1 superficial infection (0.75%) requiring debridement were observed.

Comparatively, there was no statistically significant difference between the two groups regarding HHS and hip range of motion ( Table 2 ). The number of cases of iliopsoas tendinitis, heterotopic ossification, and readmission due to complications was higher in group 2.

**Table 2:** Harris Hip Score (HHS) and range of motion in groups 1 and 2

Variable	Group 1–mean (minimum–maximum)	Group 2–mean (minimum–maximum)
**Preoperative HHS**	52.5 (41.5–62.5)	52.4 (41.0–62.75)
**HHS at 3 months**	92.5 (80.7–99.9)	92.4 (77.9–99.9)
**HHS at the last follow-up**	97.3 (94.6–99.9)	97.0 (94.2–100.0)
	≤ 0.001*	≤ 0.001*
**Preoperative flexion (degrees)**	68.9 (50.0–90.0)	69.7 (50.0–90.0)
0.714**		
**Preoperative external rotation (degrees)**	6.0 (0.0–15.0)	7.2 (0.0–20.0)
0.097**		
**Flexion at the last follow-up (degrees)**	100.0 (90.0–110.0)	100.3 (90.0–110.0)
0.448**		
**External rotation at the last follow-up (degrees)**	32.0 (10.0–45.0)	34.0 (5.0–45.0)
		0.184**

**Notes:** *Friedman test; **Mann-Whitney U test. **Group 1** : Primary total hip arthroplasty using the direct anterior approach with capsular preservation and closure. **Group 2** : Primary total hip arthroplasty using the direct anterior approach with resection of the articular capsule (capsulectomy).

## Discussion

In the prospective cohort currently described, the main surgical complications were heterotopic ossification and iliopsoas tendinitis. The results demonstrate the importance of the joint capsule as an anatomical protector between the acetabular prosthetic component and the iliopsoas.

Although there was no significant difference in dislocation rates between our groups, a systematic review of 31 articles by Miranda et al. 13 reported that patients undergoing capsular repair had a lower dislocation rate (0.65%) than those undergoing capsulectomy (3.06%). In another study, Kanda et al. 14 found no evidence of prosthesis dislocation in patients with preserved joint capsules during a 6-month follow-up. Imagama et al. 15 demonstrated that anterior capsular suture reduces axial traction force produced by the surgeon intraoperatively.

In contrast, Vandeputte et al., 16 in a prospective randomized cohort study with 219 hips comparing capsular preservation and resection techniques, concluded that there was no statistically significant difference between the variables analyzed.

McLawhorn et al. 17 conducted a prospective study involving 32 patients, of whom 17 underwent capsular repair and 15 underwent capsulectomy. At the 1-year follow-up, magnetic resonance imaging demonstrated an intact anterior capsule in patients who underwent capsular repair, whereas a persistent capsular defect was observed in 27% of patients who underwent capsulectomy.

## Conclusion

Performing capsule preservation and closure in THA using DAA resulted in a lower incidence of clinical and radiographic complications, particularly regarding iliopsoas tendinitis and heterotopic ossification, when compared with the capsulectomy group. Joint function, assessed by the HHS, was similar in both groups.
